# Supplementary material for: Comparative genomics provides new insights into the diversity, physiology, and sexuality of the only industrially exploited tremellomycete: Phaffia rhodozyma
Source: BMC Genomics. 2016 Nov 9;17:901. doi: 10.1186/s12864-016-3244-7 (PMC5103461; doi:10.1186/s12864-016-3244-7)
Supplement: Additional file 6: — List of orphan genes with links to PFAM (related to Additional file 1: Table S1). (ZIP 1428 kb) [file 12864_2016_3244_MOESM6_ESM.zip › PFAM_UNIQUE/G01971_P.html]

#                                                                            --- full sequence --- -------------- this domain -------------   hmm coord   ali coord   env coord
  
# target name        accession   tlen query name           accession   qlen   E-value  score  bias   #  of  c-Evalue  i-Evalue  score  bias  from    to  from    to  from    to  acc description of target
  
#------------------- ---------- ----- -------------------- ---------- ----- --------- ------ ----- --- --- --------- --------- ------ ----- ----- ----- ----- ----- ----- ----- ---- ---------------------
  
G01971\_P             -            163 ASD2                 PF08687.6    264       5.4    4.9   8.9   1   1     0.013       7.6    4.4   8.3    16   135    35   158    18   161 0.61 - Apx/Shroom domain ASD2  
G01971\_P             -            163 BAG                  PF02179.11    76   0.00014   20.5   2.3   1   2   1.1e-07   0.00022   20.0   0.5    29    69    84   127    58   128 0.84 - BAG domain  
G01971\_P             -            163 BAG                  PF02179.11    76   0.00014   20.5   2.3   2   2      0.59   1.2e+03   -1.6   0.0     2    16   146   160   145   161 0.80 - BAG domain  
G01971\_P             -            163 FliT                 PF05400.8     84       1.5    8.0  13.6   1   2   5.4e-05      0.11   11.6   5.6     8    64    21    79    14    88 0.80 - Flagellar protein FliT  
G01971\_P             -            163 FliT                 PF05400.8     84       1.5    8.0  13.6   2   2      0.13   2.5e+02    0.8   0.9    17    40   116   145   106   160 0.47 - Flagellar protein FliT  
G01971\_P             -            163 Nucleo\_P87           PF07267.6    654       1.2    6.3   8.4   1   1    0.0048       1.7    5.9   8.4   320   445    21   151     6   157 0.67 - Nucleopolyhedrovirus capsid protein P87  
G01971\_P             -            163 PRCC                 PF10253.4    182       3.1    7.1   6.7   1   1    0.0083       4.9    6.5   6.7    16   119    14   121     3   159 0.52 - Mitotic checkpoint regulator, MAD2B-interacting  
G01971\_P             -            163 Retinal              PF15449.1   1287       4.8    3.4   6.5   1   1     0.016       6.2    3.0   6.5   469   528     4    63     1    74 0.62 - Retinal protein  
G01971\_P             -            163 SGT1                 PF07093.6    589       8.1    3.3  13.7   1   1     0.055        10    3.0  13.7   371   520    13   154     4   160 0.76 - SGT1 protein
